# The Oncological Value of Omentectomy in Gastrectomy for Cancer

**DOI:** 10.1007/s11605-016-3092-4

**Published:** 2016-02-19

**Authors:** Leonie Haverkamp, Hylke J. F. Brenkman, Jelle P. Ruurda, Fiebo J. W. ten Kate, Richard van Hillegersberg

**Affiliations:** Department of Surgery, University Medical Center Utrecht, G04. 228, PO 85500, 3508 GA Utrecht, The Netherlands; Department of Pathology, University Medical Center Utrecht, PO 85500, 3508 GA Utrecht, The Netherlands

**Keywords:** Omentum, Lymph nodes, Gastric cancer

## Abstract

**Aim:**

The aim of this study was to determine the oncologic value of omentectomy in patients undergoing gastrectomy for gastric cancer.

**Methods:**

All consecutive patients with gastric cancer that underwent gastrectomy with curative intent between April 2012 and August 2015 were prospectively analyzed. The greater omentum was separately marked during operation and pathologically evaluated for the presence of omental lymph nodes and tumor deposits.

**Results:**

In total, 50 patients were included. The greater omentum harbored lymph nodes in nine (18 %) patients. The omental lymph nodes contained metastases in one (2 %) patient, still free of disease after 20 months. Omental tumor deposits were found in four (8 %) patients; one died <30 days postoperative and three developed peritoneal carcinomatosa after 4, 4, and 8 months. Patients with omental tumor deposits had a significantly reduced 1-year disease-free survival compared to patients without tumor deposits (0 vs. 58.7 %, *p* = 0.003). No predictive factors for omental tumor involvement could be identified.

**Conclusion:**

Omental lymph node metastases or tumor deposits are present in 10 % of Western European patients undergoing gastrectomy for gastric cancer. Omentectomy has a prognostic and oncologic value in the curative treatment of patients with gastric cancer. As no predictive factors for omental tumor involvement could be identified, omentectomy should be the standard in gastrectomy for gastric cancer patients.

## Introduction

The standard of care for the curative treatment of gastric adenocarcinoma is surgical resection with perioperative chemotherapy.^1^^,^^2^ The oncologic importance of omentectomy during total and distal gastrectomy remains unclear. The European, American, and Japanese guidelines for the treatment of gastric adenocarcinoma have not reached uniform consensus with respect to omentectomy. The European guidelines do not give any advice regarding omentectomy,^3^^,^^4^ whereas the most recent American guidelines advise to resect both the greater and lesser omentum.^5^ Alternatively, the Japanese Gastric Cancer Association recommends preservation of the greater omentum at >3 cm from the gastroepiploic arcade for patients with T1-T2 tumors and total omentectomy for patients with T3-T4 tumors.^6^ The inconsistency between guidelines on this topic reflects the lack of studies that evaluate the patterns of lymphatic spread and omental tumor involvement during primary curative surgery for gastric cancer.

The function of the greater omentum is to localize and encapsulate the sites of infection to limit intra-abdominal disease.^7^^,^^8^ Omentectomy is an additional surgical procedure to gastrectomy, which may lead to longer operation time and might add to morbidity.^8^^–^10 The aim of this study was to evaluate the value of the resection of the greater omentum in Western patients that underwent gastrectomy with omentectomy at curative intent for gastric adenocarcinoma.

## Material and Methods

### Patients

All consecutive patients who underwent a curative gastrectomy with omentectomy for gastric adenocarcinoma at the University Medical Center Utrecht between April 2012 and August 2015 were included. In this prospective cohort, the gastrocolic ligament and the actual greater omentum were dissected and marked separately during surgery. The greater omentum was divided in four quadrants for pathological evaluation of the patterns of lymphatic spread and tumor deposition. The surgical resection and marking and the pathological processing and analysis of the specimen were performed according to a standardized protocol. Clinical data were retrieved from the medical records. Survival status was evaluated during follow-up and additionally by contacting the patient’s general practitioner and the municipal personal records. The study was performed according to institutional ethical review board requirements.

### Surgical Technique

The indications for total gastrectomy were tumor location proximal from the antrum and linitis plastica. Distal gastrectomy was performed in tumors located in the antrum that could be resected with an adequate 5-cm resection margin. All patients underwent a laparoscopic resection. All patients underwent an intra-abdominal inspection before gastrectomy commenced. When abdominal tumor deposits were seen, patients were not included in this study as no curative resection was performed. Patients underwent en bloc resection of the stomach and the lesser omentum, combined with a D2 lymph node dissection.^6^ The greater omentum was dissected at the distal side of the gastroepiploic artery through the avascular plane connecting it to the transverse colon. The omentectomy involved the entire width of its attachment to the transverse colon. After separate removal, the greater omentum was marked at four quadrants (left, right/cranial, caudal) during surgery. Neither pancreatectomy nor splenectomy was performed routinely. Perioperative chemotherapy (epirubicin, cisplatin, and fluorouracil) was administered perioperatively in patients with >cT1 or >cN1 disease when considered possible based on comorbidity and performance status.^2^

### Pathological Analysis

After operation, the omentum was fixed in 4 % formaldehyde for at least 12 h. After fixation, the omentum, marked by the surgeons in four quadrants, was dissected in lamella of 1–2 cm and macroscopically evaluated by an experienced GI pathologist (FJWK) for the presence of lymph nodes or tumor deposits. Potential tumor deposit or lymph nodes were isolated and sampled. In case no tumor depositions or lymph nodes were found, at least one ad random sample per quadrant was taken. To extract lipids, all samples were incubated in acetone for 8 h. Subsequently, the samples were routinely processed for paraffin embedding, using the vacuum infiltration processor VIP 5. From the paraffin blocks, 4-μm sections were stained with hematoxylin and eosin (H&E). In difficult cases, immunohistochemical stainings were performed with pan-keratin marker CKAE1/3 (LabVision/Neomarkers, Fremont, CA, USA) and the epithelial tumor marker CAM 5.2 (Becton-Dickins, Franklin Lakes, NJ, USA).

### Statistical Analysis

Statistical analysis was performed with the use of SPSS (version 20.0, SPSS Inc, Chicago, IL, USA, 2011). The Pearson *χ*^2^ was used for binary and categorical values, whereas the nonparametric Mann-Whitney *U* test was used to analyze continuous variables with a non-Gaussian distribution. Binary regression analysis was performed for multivariate analysis. All covariables with a *p* value < 0.20 in univariate analysis were included for multivariate analysis. A Kaplan-Meier curve was constructed to analyze cumulative 1-year survival. The log rank (Mantel-Cox) was used to calculate the significance. *P* values < 0.05 were considered to indicate statistical significance; 95 % confidence intervals were used. Data were presented as median with range between brackets.

## Results

### Patient Characteristics

A total of 50 patients with gastric adenocarcinoma underwent gastrectomy with omentectomy and were included. Their median age was 68 (40–87) years and 26 (52 %) were male (Table 1). Total gastrectomy was performed in 41 (82 %) patients, whereas distal gastrectomy was performed in 9 (18 %) patients. The tumor was located in the gastric cardia in 22 (44 %) patients and in the antrum in 12 (24 %) patients (Table 1). The majority of patients (*n* = 32, 64 %) were diagnosed with cT3 gastric cancer. Chemotherapy was administered to 35 (70 %) patients preoperatively.

**Table 1:** Baseline characteristics

	*N* = 50
Age (years)^a^	68 (40–87)
Gender	
Male	26
Female	24
Type of surgery	
Total gastrectomy	41
Distal gastrectomy	9
Site of tumor	
Cardia	22
Corpus	7
Antrum	12
Pre-pyloric	1
Pylorus	1
Diffuse	7
Tumor differentiation grade	
Not assessed	3
Well	7
Moderate	13
Poor	27
Primary tumor, cT	
1	5
2	13
3	32
Regional lymph nodes cN	
0	24
1	11
2	13
3	2
Clinical stage	
IA	3
IB	10
IIA	16
IIB	8
IIIA	11
IIIB	2
Preoperative chemotherapy	35 (70 %)

^a^Data presented as median (minimum-maximum)

### Pathological Analysis

**Fig. 1:**
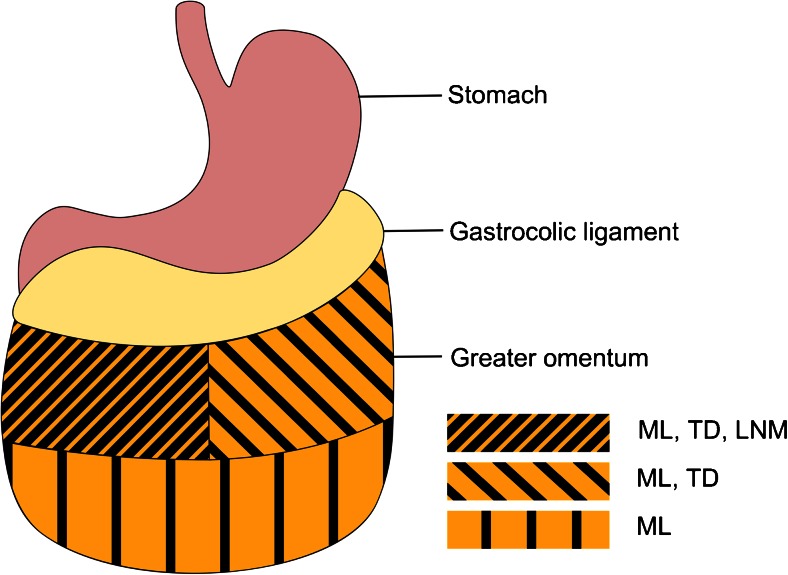
Location of tumor deposits (TD), lymph node metastases (LNM), and multifocal locations of tumor deposits (ML) in the greater omentum in five patients

In 47 (94 %) patients, a radical resection (R0) of the tumor was achieved (Table 2). The median length of the greater omentum was 32 (14–60) cm and the median width was 15 (5–40) cm. The median number of lymph nodes in the resected specimen was 19 (5–62) (Table 3). Lymph nodes in the greater omentum were found in nine (18 %) patients, of which one (2 %) patient with stage IB was positive for tumor cells. No perioperative chemotherapy was administered to this patient. Tumor deposits of gastric carcinoma were found in the greater omentum in four (8 %) patients, clinical stage IB (*n* = 1), IIA (*n* = 1), IIB (*n* = 1), and IIIA (*n* = 1). Perioperative chemotherapy was given in 3/4 patients. The lymph node metastasis was located in the right cranial quadrant of the omentum and the primary tumor was located in the antrum. The locations of the tumor deposits were right cranial (*n* = 1), left cranial (*n* = 1), and multifocal (all quadrants, *n* = 2) (Fig. 1). The locations of the primary tumors were diffuse (*n* = 2), corpus (*n* = 1), and cardia (*n* = 1). In univariate and multivariate analyses, no significant risk factors, such as advanced tumor stage, for the presence of omental lymph node metastases and tumor deposits were identified (Table 4).

**Table 2:** Oncologic outcomes

	*N* = 50
Radicality	
R0	47
R1	3
Recurrent disease	20
Local	8
Peritoneal	13
Distant	6

**Table 3:** Pathological outcomes

	*N* = 50
Overall	
Number of lymph nodes^a^	19 (5–62)
Patients with N+	29
Number of lymph node metastases in N+ patients^a^	4 (1–12)
Greater Omentum	
Patients with omental lymph nodes	9
Patients with omental lymph node metastases	1
Omental tumor deposits	4

^a^Data presented as median (minimum-maximum)

**Table 4:** Risk factors for omental lymph node metastases or tumor deposits

Characteristic	Univariate		Multivariate	
	Odds ratio (95 % CI)	*p*	Odds ratio (95 % CI)	*p*
Tumor location				
Cardia, fundus	0.286 (0.030–2.761)	0.279	0.394 (0.029–5.439)	0.487
Diffuse	5.333 (0.710–40.057)	0.104	3.903 (0.386–39.456)	0.249
Clinical Stage III	0.667 (0.068–6.580)	0.729	1.415 (0.105–19.012)	0.793
Preoperative chemotherapy	0.609 (0.091–4.081)	0.610	1.019 (0.122–8.528)	0.986
Total versus distal gastrectomy	1.156 (0.114–11.774)	0.902		
Poor tumor differentiation	1.125 (0.170–7.452)	0.903		

*95 %* *CI 95 %* confidence interval

### Survival

After a median follow-up of 9 (0–42) months, 20 (40 %) patients developed recurrent disease. Local recurrence of the gastric adenocarcinoma occurred in 8 (16 %) patients, peritoneal metastases occurred in 13 (26 %) patients, and distant metastases were found in 6 (12 %) patients (Table 2). A combination of local recurrence and peritoneal/distant metastases was found in 7 (14 %) patients. The median time to recurrent disease was 8 (1–17 months. Of the five patients with omental tumor involvement, one patient with omental tumor deposits died within 30 days postoperative. The patient with the positive omental lymph node was still free of disease after 20 months. In the patients with a follow-up >30 days, peritoneal recurrence was seen in all three patients with omental tumor deposits, compared to 10/45 patients without omental tumor deposits (100 % vs. 22 %, *p* = 0.017). The three patients with omental tumor deposits developed peritonitis carcinomatosa 4, 4, and 8 months after surgery resulting in a reduced 1-year disease-free survival compared to patients without omental tumor deposits (0.0 vs. 58.7 %, respectively, *p* = 0.003). No significant difference in 1-year overall survival of patients with and without omental tumor deposits was found (25.0 vs. 67.4 %, respectively, *p* = 0.079).

## Discussion

This is the first study that prospectively investigated the presence of lymph node metastases and tumor deposits in the greater omentum in a series of Western European patients with gastric adenocarcinoma undergoing gastrectomy. This study shows an oncologic value of omentectomy, since the greater omentum harbored lymph nodes in eight (16 %) patients and lymph node metastases were found in the greater omentum in one (2 %) patient. Secondly, this study shows a prognostic value of omentectomy. The greater omentum harbored tumor deposits in four (8 %) patients, of which none were free of disease 1 year after surgery, compared to 59 % of patients without omental tumor deposits. No clinical or pathological factors were significantly associated with omental tumor involvement.

The greater omentum has an important function in localizing and encapsulating sites of infection to limit intra-abdominal disease. Resection of the greater omentum is an additional burden to the patient. It might be argued that leaving the omentum in place would be beneficial, especially in laparoscopic gastrectomy, aiming at a minimally invasive procedure and reduced morbidity with normal physiology maintained. On the other hand, leaving the greater omentum in place may cause omental infarction. Indeed, a radiologic study showed omental infarction on postoperative CT scans in 9/390 (2.3 %) of patients who underwent laparoscopy-assisted gastrectomy with partial omentectomy for T1-T2 gastric cancer. However, these infarctions resolved over time in all patients and did not have clinical consequences.^11^ In a retrospective study of patients that underwent gastrectomy for early gastric cancer, no significant difference in early complication rate was found between the group with and without omentectomy (3.5 vs. 3.2 %). In contrast, late complications, defined as occurring after discharge or from 30–60 days after surgery, occurred significantly less in the omentum-preserving group (3.5 vs. 0 %; *p* = 0.026). The described late complications were intra-abdominal abscesses, ileus, mechanical bowel obstruction, and anastomotic leakage. No differences regarding mortality were reported.^8^

A clinical study in Asian patients (*n* = 196) with advanced stage gastric cancer found that there was no significant difference in 3- and 5-year survival rates between gastrectomy with and without omentectomy.^12^ In our study, 10 % of the patients showed tumor involvement in the greater omentum. The patient with the positive lymph node in the right cranial quadrant of the greater omentum was still free of disease after 20 months. These findings support the additive oncologic value for an adequate omentectomy. None of the patients with omental tumor depositions was free of disease 1 year after surgery, compared to 59 % of patients without omental tumor deposits (*p* = 0.003). Since all patients with omental tumor deposits developed peritonitis carcinomatosa, this subgroup of patients might benefit from additional treatment, such as hyperthermic intraperitoneal chemotherapy (HIPEC), which may be tested in future studies. To identify patients with omental tumor involvement for additional treatment, a thorough pathological evaluation of the greater omentum should be performed along with the gastrectomy.

The strength of our current series is that the greater omentum was specifically marked during surgery for this study to allow for meticulous pathological evaluation of each quadrant. In this way, the patterns of lymphatic spread could be very reliably analyzed. In the current prospective patient series, we deliberately made clear distinction between the gastrocolic ligament and the actual greater omentum, also referred to as infracolic omentum. To objectively evaluate the infracolic omentum, the greater omentum was dissected distal to the gastroepiploic artery and removed separately. Results on omental lymph node counts may otherwise reflect the perigastric lymph nodes status, rather than those located in the greater omentum. As the omentum was resected separately and marked during surgery, we could very precisely locate the lymph node metastases and tumor deposits exclusively in the greater omentum. The lymph node metastases were found in the right cranial quadrant. The primary tumor was located in the antrum, which might explain the local spread. The tumor deposits were found in the left and right cranial quadrants and multifocally throughout the omentum. The location of the primary tumor did not influence the location of the tumor deposits. The multifocal spread is in accordance with the current understanding of the lymphatic drainage of the omentum by means of milky spots, which are located throughout the entire greater omentum.^13^ These are sites of macrophages and lymphocytes located around capillary convolutions and drain peritoneal fluid into lymph collectors that empty into subpyloric nodes.^8^^,^^13^ Seeding of gastro-intestinal cancer cells preferentially occurs to the milky spots.^8^

There is no consensus worldwide on whether to perform an omentectomy or not during gastrectomy. The European guidelines do not give any advice regarding omentectomy,^3^^,^^4^ whereas the most recent American guidelines advise to resect both the greater and lesser omentum.^5^ According to the Japanese guidelines for gastric cancer, the greater omentum can be left in place in the specific subgroup of patients with T1 and T2 tumors. However, our study with Western European patients shows the presence of omental lymph node metastases and tumor deposits also in patients with early gastric cancer. Since no predictive factors were associated with the presence of lymph node metastases or tumor deposits in the greater omentum, we advise an omentectomy in all patients undergoing gastrectomy.

## Conclusion

In conclusion, omental lymph node metastases and tumor deposits are found in a substantial part of Western European patients undergoing gastrectomy for gastric cancer. Omentectomy has a prognostic and oncologic value in the curative treatment of patients with gastric cancer. An omentectomy should be performed in all patients with resectable gastric cancer, since no predictive factors were associated with omental tumor involvement.
